# The Evolution of Clinicopathological Diagnostic Features of Upper Tract Urothelial Carcinoma in China: A Summary of 2561 Cases in the Last 20 Years

**DOI:** 10.3389/fonc.2022.769252

**Published:** 2022-03-09

**Authors:** Chunru Xu, Changwei Yuan, Cuijian Zhang, Dong Fang, Yanfei Yu, Xiang Wang, Zhihua Li, Yan Wang, Qi Tang, Gengyan Xiong, Lei Zhang, Zhisong He, Jian Lin, Liqun Zhou, Xuesong Li

**Affiliations:** ^1^ Department of Urology, Peking University First Hospital, Beijing, China; ^2^ Institute of Urology, Peking University, Beijing, China; ^3^ National Urological Cancer Center, Beijing, China; ^4^ Department of Nursing, Peking University First Hospital, Beijing, China

**Keywords:** evolution, clinicopathological diagnostic features, upper tract urothelial carcinoma (UTUC), Chinese population, 20 years

## Abstract

**Objectives:**

To summarize the clinicopathological diagnostic features and evolutionary trends of upper tract urothelial carcinoma (UTUC) in China over the past 20 years.

**Methods:**

All patients diagnosed with upper tract urothelial carcinoma in the Peking University First Hospital from 2001 to 2020 were retrospectively collected. Data were divided into two groups (2001-2010 and 2011-2020) according to the date of diagnosis. Statistical analysis was done with the SPSS V22.0. Chi-square analysis and t-test were adopted to analyze depending on the data type. Subgroup analysis based on 5 years was used for visualization to present trends. Both Kaplan-Meier curve and Cox regression were used for univariate and multivariate survival analysis.

**Results:**

The study included 2561 cases diagnosed with upper tract urothelial carcinoma in total. Compared with the first decade (2001-2010), patients of the second decades (2011-2020) had elder mean age (66.65 versus 67.59, years, p=0.025), higher male proportion (43.5% versus 49.0%, p=0.034), lower incidence of renal pelvic tumors (53.4% versus 45.8%, p<0.001) and multifocality (18.6% versus 12.0%, p<0.001), higher incidence of ureteral tumors (52.2% versus 60.9%, p<0.001).In recent ten years, the incidence of muscle-invasive urothelial carcinoma (pT2+) decreased significantly (64.4% versus 54.9%, p<0.001),and the mean size of renal pelvic tumors increased(3.46 versus 3.73, cm, p=0.043). The size of the ureteral tumor, the histopathologic grade showed no significant change. The prognostic analysis based on 709 patients regularly followed at our center revealed that the male gender and G3 histopathological grade were independent risk factors for poorer prognosis in patients with UTUC.

**Conclusion:**

In the past 20 years, the clinicopathological diagnostic features of upper tract urothelial carcinoma in the Chinese population has changed significantly, suggesting an increased risk of a poorer prognosis for UTUC. This trend may be related to updating diagnostic techniques and self-monitoring awareness. However, we need more high-grade, multicenter trials to verify it in the future.

## Introduction

Upper tract urothelial carcinoma (UTUC) is an uncommon malignant disease that occurs in the pyelocaliceal cavity and ureter, accounting for only 5% to 10% of the urothelial carcinomas (UCs) (1). UTUCs usually were multicentric and prone to recurrence, and have a higher grade and stage at diagnosis (2). Therefore, the early diagnosis and management of UTUC have been a hot issue of clinical concern.

A recent study based on the SEER database showed that the incidence of UTUC in the United States gradually declined over the last 30 years and that the disease was significantly more prevalent in people over 70 years of age, in men, and renal pelvis (3). However, presumably due to the widespread application of aristolochic acid drugs, UTUC in the Chinese populations has different epidemiological characteristics from those of Western populations. Compared to Western people, the Chinese UTUC population has a higher tumor grade, relatively lower tumor stage, and lower malignancy in female patients compared to the males. And there were more female patients than their counterparts (4). To our knowledge, there was no study on the trend of evolution of the pathological characteristics of the UTUC of the Chinese population in recent years.

This study aimed to summarize the clinical and pathological characteristics of UTUC of the Chinese population based on large sample size and to explore the association between pathological features and clinical characteristics of the UTUC. We further analyzed and elucidated the evolution of the distribution of pathological and clinical features of UTUC in the Chinese population over the past 20 years.

## Patients and Methods

### Patient Selection

All consecutive UTUC patients who underwent radical nephroureterectomy or segmental ureterectomy or only ureteroscopy in Peking University First Hospital, one of the largest urological clinical centers in China with a large number of UTUC patients from all over China and the world each year, between January 2001 and December 2020, were included in the first screening process. The HIS (Hospital Information System) was the help in collecting basic patient information and pathology information. Cases with a pathological diagnosis of non-UTUC, cytological findings only, history of neoadjuvant chemotherapy, and duplicates were filtered and excluded by two researchers (CR Xu and CW Yuan). The protocol of this study was approved by the Ethics Committee of Peking University First Hospital. Approval reference number. 2021[130].

### Pathological Diagnosis

Tumors were staged by the 2002 TNM classification system (5) and graded as G1, G2, and G3 according to the 1973 WHO classification system (6) (Considering that some pre-2004 cases have not yet adopted 2004 WHO updated grade criteria). Other pathological information such as tumor multifocality, primary location (renal pelvic or ureter), and the maximum diameter of the mass were analyzed as well. When masses are found in the renal pelvis and ureter separately at the same time, the primary sites were recorded separately. Each pathological diagnosis was made by two pathologists who independently reviewed and agreed on their conclusions, and in the event of disagreement between the two physicians, the diagnosis was reviewed by another higher-level pathologist.

### Prognostic Information and Analysis

To further investigate the association of pathological features with patients’ prognosis and quality of survival, we collected a total of 709 unselected patients with UTUC who had previously received regular follow-up from 2001 to 2021 at our center and for whom complete prognostic survival data were available (all included in the overall sample of this study). Basic patient information and pathological characteristics such as gender, tumor location, multifocality or not, presence of muscle invasion, and pathological grade were included in the analysis. Cancer-specific survival (CSS) was adopted as the primary prognostic endpoint.

### Statistical Analysis

All data were divided into two groups according to the time of diagnosis at every ten years (2001-2010 and 2011-2020). To better show the trends in pathological characteristics over time, subgroup analyses made senses according to 5 years (Shown in **Tables 2-B**, **3-B**). For categorical variables, we applied the R*C columnar combined chi-square method to analyze the differences in the comparison rates. The t-test, on the other hand, was suitable for the comparison of sample means of continuous type variables. The results of Fisher’s exact test were adopted when the expectation value <5 appeared in the results of the R*C column table. Kaplan-Meier curve and Cox regression were used for prognostic analyses, and variables satisfying p < 0.2 after univariate analysis were selected for inclusion in the multivariate regression test. All statistical analysis processes were done with the help of SPSS V22.0 (IBM, Armonk, NY). P values are 2-sided, with statistical significance defined as P<0.05. Graphpad Prism version 8.0.1 (GraphPad, San Diego) was used to visualize data. Two authors (CR Xu and CW Yuan) separately completed the data input and statistical analysis, with the entire process supervised by a third author (XS Li).

## Results

From 2001 to 2020, a total of 3269 patients recorded were found in the His system. After removing duplicate records, records of non-surgical treatments, and data with cytological pathology results only, 2561 cases of UTUC were finally recruited in this study. The clinicopathological characteristics of these patients, including 1214 (47.4%) males and 1347 (52.6%) females, with 1228 (48.0%) tumors of renal pelvic, 1496 (58.4%) ureteral tumors, and 356 (13.9%) multifocal tumors were summarized in **Table 1**.

**Table 1:** Clinicopathological characteristics of included patients.

Variables	Patients, n	Median (IQR) or %
Age, years	2561	68 (20-93)
<55	272	10.6%
55-60	1352	52.8%
≥70	937	36.6%
Gender		
Male	1214	47.4%
Female	1347	52.6%
Location		
Renal Pelvic	1228	48.0%
Ureter	1496	58.4%
Multifocality	356	13.9%
With Cis	69	2.7%
T Stage of all cases		
T Not clear	20	0.8%
Cis only	2	0.1%
T1+Ta	1063	41.5%
T2	780	30.5%
T3	647	25.3%
T4	49	1.9%
Grading of all cases		
G1	58	2.3%
G2	1369	53.5%
G3	1134	44.3%
Tumor of Renal Pelvic		
T Not clear	6	0.5%
Cis only	1	0.1%
T1+Ta	517	42.1%
T2	287	23.4%
T3	379	30.9%
T4	37	3.0%
G1	14	1.1%
G2	759	61.9%
G3	455	37.1%
Tumor Size (maximum, cm)		3.2 (0.1-27.0)
<2.0cm	192	15.6%
2-5cm	763	62.1%
≥5.0cm	273	22.2%
Tumor of Ureter		
T Not clear	18	1.2%
Cis only	3	0.2%
T1+Ta	609	40.7%
T2	532	35.6%
T3	322	21.5%
T4	12	0.8%
G1	46	3.1%
G2	696	46.5%
G3	754	50.4%
Tumor Size (maximum, cm)		2.5 (0.1-27.0)
<2.0cm	373	24.9%
2-5cm	954	63.8%
≥5.0cm	169	11.3%

Cis, carcinoma in situ.

### Age, Gender and Primary Site of Tumors

**Table 2-A** demonstrated the changes in age, gender composition, and primary site of the UTUC patients in the last 20 years. Comparing the 1st decade (2001 to 2010, abbreviated as 1stD) versus the 2nd decade (2011 to 2020, abbreviated as 2ndD), the age of UTUC patients showed a significant increasing trend (p=0.025), but both the proportion of the young patients (age<55 years old) and elderly patients group (age≥70 years old) did not change significantly (p=0.088; p=0.853). In terms of gender composition, there were more female patients than male patients in the last 20 years (1347 versus 1214, 1.11:1). The difference in sexual ratio decreased over the past ten years, while the proportion of male patients significantly rise (p=0.034).

**Table 2-A:** Changes in age, gender composition, and primary site of the UTUC patients in the last 20 years.

Variables	2001-2010	2011-2020	P Value
Cases	734	1827	
Gender			
Male	319 (43.5%)	895 (49.0%)	0.034^*^
Female	415 (56.5%)	932 (51.0%)
Age,years	66.65±10.11	67.59±10.09	0.025^*^
<55	90 (12.3%)	182 (10.0%)	0.088
≥70	324 (44.1%)	815 (44.6%)	0.83
Primary Site
Renal pelvic	390 (53.1%)	836 (45.8%)	<0.001^**^
Ureter	381 (53.0%)	1113 (60.9%)	<0.001^**^
Multifocality	136 (18.6%)	220 (12.0%)	<0.001^**^

*P<0.05; **P<0.01.

There was also a significant difference in the proportion of the primary site of tumors between 1stD and 2ndD. Renal pelvic tumors were found less than the last time (p<0.001), while the incidence of ureteral tumors has gradually increased (p<0.001). Specifically, the incidence of multifocal tumors decreased significantly in the recent ten years (p<0.001).

**Figure 1:**
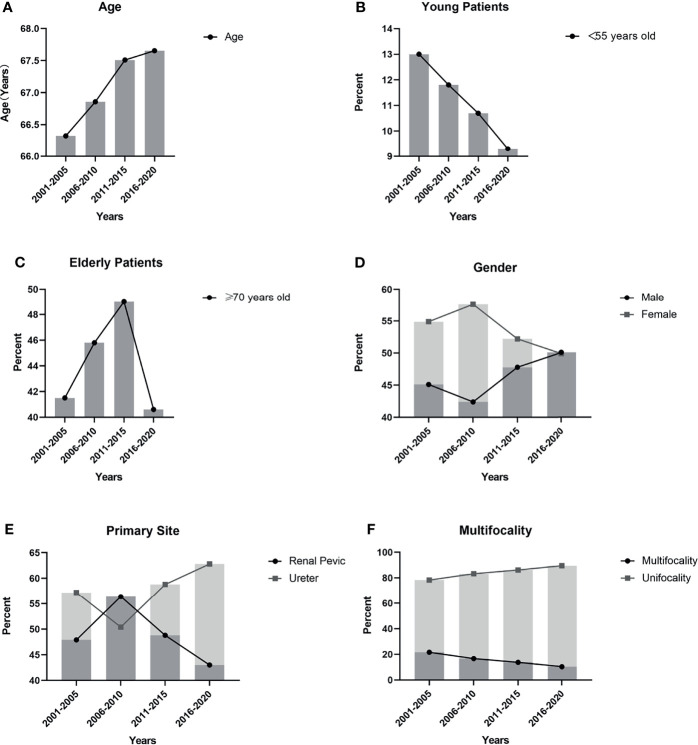
Trends in clinical characteristics based on 5 years: **(A)** The mean age of the UTUC patients gradually increased and the difference between two decades was significant (p=0.025); **(B)** The incidence of young patients (<55 years old) showed a gradual decrease, but there was no significant difference (p=0.245); **(C)** There was a significant difference (p=0.002) in the incidence rate of elderly patients (≥70 years old), but the trend fluctuated dramatically; **(D)** The proportion of male patients gradually declined, and the proportion of female patients gradually increased. The difference between two decades was significant (p=0.034); **(E)** The incidence of renal pelvic tumors and ureteral tumors and the difference between two decades was significant (p<0.001); **(F)** The trend showed the incidence of multifocal tumors gradually decreased and the difference between two decades was significant (p<0.001).

To better show trends in change, we further completed a five-year-based subgroup analysis. The results were broadly consistent with the ten-year-based results, with the difference that the incidence of elderly patients (≥70 years old) showed significant between-group variability (p=0.002), but the overall trend showed fluctuations. The results and trends were shown in **Table 2-B** and **Figure 1**.

**Table 2-B:** Five-year-based subgroup analysis of changes in age, gender composition, and primary site of the UTUC.

Variables		2001-2005	2006-2010		2011-2015	2016-2020	P Value	
Cases		284	450		869	958		
Gender								
Male		128 (45.1%)	191 (42.4%)		415 (47.8%)	480 (50.1%)	0.048*	
Female		156 (54.9%)	259 (57.6%)		454 (52.2%)	478 (49.9%)		
Age,years		66.32±9.63	66.86±10.40		67.51±10.39	67.65±9.82	0.168	
<55		37 (13.0%)	53 (11.8%)		93 (10.7%)	89 (9.3%)	0.245	
≥70		118 (41.5%)	206 (45.8%)		426 (49.0%)	389 (40.6%)	0.002**	
Primary Site								
Renal pelvic		137 (48.2%)	255 (56.7%)		424 (48.8%)	412 (43.0%)	<0.001**	
Ureter		156 (54.9%)	227 (50.4%)		511 (58.8%)	602 (62.8%)	<0.001**	
Multifocality		61 (21.6%)	75 (16.7%)		120 (13.8%)	100 (10.4%)	<0.001**	

*P<0.05; **P<0.01.

### Tumor Stage and Histological Grading

We detailed the trends in tumor histologic T staging and grading in **Tables 3-A** **,** **B**.

**Table 3-A:** Changes in pathological characteristics of UTUC in the last 20 years.

Variables		2001-2010	2011-2020			P Value		
Tumor of Renal Pelvic								
T Not clear		5 (1.3%)	0 (-)			<0.001^**^		
Cis		1 (0.3%)	0 (-)					
T1+Ta		125 (32.1%)	392 (46.9%)					
T2		130 (33.3%)	157 (18.8%)					
T3		120 (30.8%)	259 (31.0%)					
T4		9 (2.3%)	28 (3.3%)					
G1		6 (1.5%)	6 (0.7%)			0.385		
G2		242 (62.1%)	517 (61.8%)					
G3		142 (36.4%)	313 (37.4%)					
Tumor Size (maximum,cm)		3.46±1.97	3.73±2.15			0.043^*^		
Tumor of Ureter								
T Not clear		13 (3.4%)	6 (0.5%)			0.018^*^		
Cis		0 (-)	3 (0.3%)					
T1+Ta		136 (35.7%)	473 (42.5%)					
T2		149 (39.1%)	381 (34.2%)					
T3		80 (21.0%)	242 (21.7%)					
T4		3 (0.8%)	8 (0.7%)					
G0		2 (0.5%)	0 (-)					
G1		10 (2.6%)	34 (3.1%)			0.195		
G2		188 (49.3%)	506 (45.5%)					
G3		181 (47.5%)	573 (51.5%)					
Tumor Size (maximum,cm)		3.00±2.47	3.08±2.29			0.609		
MIUC^a^		470 (64.0%)	1001 (54.8%)			<0.001^**^		
High-grade(G3)		310 (42.2%)	824 (45.1%)			0.187		

*P<0.05; **P<0.01; a MIUC, muscle-invasive urothelial carcinoma (pT2+).

**Table 3-B:** Five-year-based subgroup analysis of changes in pathological characteristics of UTUC.

Variables		2001-2005			2006-2010	2011-2015		2016-2020	P Value
Tumor of Renal Pelvic									
T Not clear		3 (2.2%)			3 (1,2%)	0 (-)		0 (-)	<0.001^**^
Cis		0 (-)			1 (0.4%)	0 (-)		0 (-)	
T1+Ta		33 (24.1%)			92 (36.1%)	200 (47.1%)		192 (46.6%)	
T2		45 (32.8%)			85 (33.3%)	85 (20.0%)		72 (17.5%)	
T3		52 (42.4%)			68 (26.7%)	128 (30.2%)		131 (31.8%)	
T4		4 (2.9%)			6 (2.4%)	11 (2.6%)		17 (4.1%)	
G1		7			1	4		2	<0.001^**^
G2		80			162	285		232	
G3		50			92	135		178	
Tumor Size (maximum,cm)		3.49±2.03			3.45±1.95	3.86±1.98		3.60±2.30	0.068
Tumor of Ureter									
T Not clear		3 (1.9%)			9 (4.0%)	2 (0.4%)		4 (0.7%)	<0.001^**^
Cis		0 (-)			0 (-)	1 (0.2%)		2 (0.3%)	
T1+Ta		37 (23.7%)			99 (43.6%)	222 (43.4%)		251 (41.7%)	
T2		86 (55.1%)			65 (28.6%)	171 (33.5%)		210 (34.9%)	
T3		26 (16.7%)			54 (23.8%)	112 (21.9%)		130 (21.6%)	
T4		4 (2.6%)			0 (-)	3 (0.6%)		5 (0.8%)	
G1		9 (5.8%)			3 (1.3%)	24 (4.7%)		10 (1.7%)	<0.001^**^
G2		76 (48.7%)			114 (50.2%)	250 (48.9%)		256 (42.5%)	
G3		71 (45.5%)			110 (48.5%)	237 (46.4%)		336 (55.8%)	
Tumor Size (maximum,cm)		3.09±2.16			2.95±2.67	3.07±2.04		3.09±2.48	0.901
MIUC^a^		214 (75.4%)			259 (57.6%)	475 (54.7%)		528 (55.1%)	<0.001^**^
High-grade(G3)		121 (42.6%)			189 (42.0%)	339 (39.0%)		485 (50.6%)	<0.001^**^

*P<0.05; **P<0.01; ^a^MIUC, muscle-invasive urothelial carcinoma (pT2+) in **Table 2–A**.

First, we performed a subgroup analysis to investigate whether the pathological features of MICUs (muscle-invasive urothelial carcinomas, pT2+) and high-grade (G3) UTUC were associated with the clinical features of tumors. It was found that compared with the female, a higher proportion of male patients had MIUCs (54.5% versus 61.1%, p=0.001). Young patients (<55 years old, 37.9% *vs* 45.0%, p<0.001), patients with UTUC primarily sited in the renal pelvis (37.1% *vs* 50.9%, p<0.001) had a lower incidence of high-grade (G3) tumors compared with the elder patients (≥55 years old) and patients with no renal pelvic tumor. Patients with primary ureteral tumors had a higher incidence of high-grade tumors compared to non-ureteral tumors (50.7% versus 35.2%, p<0.001).

**Figure 2:**
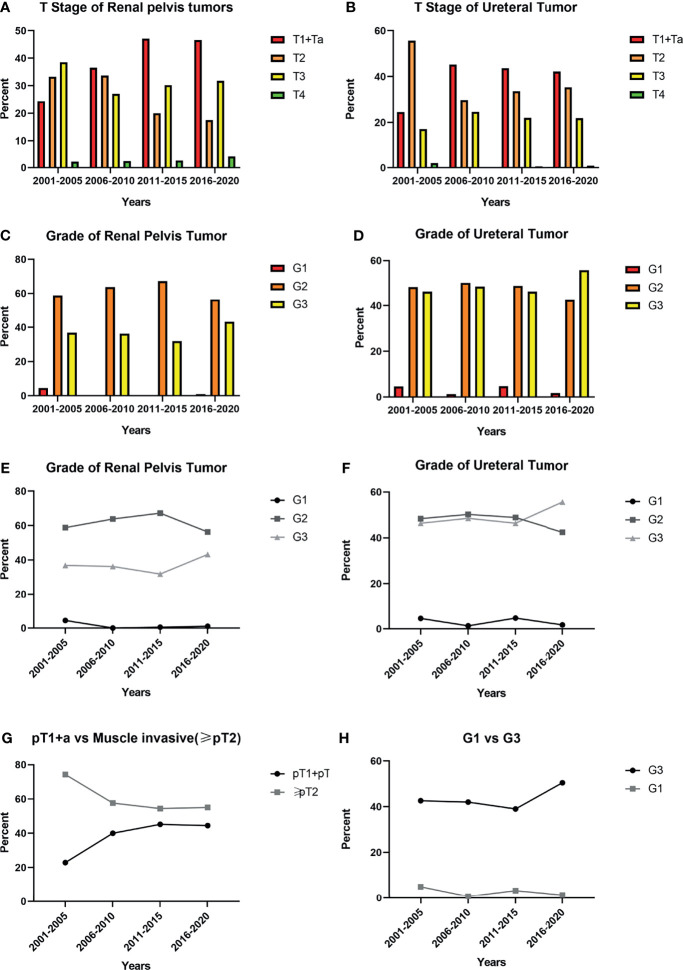
Trends in pathological characteristics based on 5 years: **(A, B)**. The incidence of different pT stage of renal pelvic tumors and ureteral tumors in previous 20 years; **(C, D)**: The incidence of different histological grading stage of renal pelvic tumors and ureteral tumors in previous 20 years; **(E, F)** The trend of incidence of different histological grading stage of renal pelvic tumors and ureteral tumors in previous 20 years; **(G)** The trend showed the incidence of muscle invasive urothelial carcinomas (MIUCs) gradually declined and the difference between two decades was significant (p<0.001); **(H)** The trend showed the incidence of high-grade(G3) UTUC gradually increased, but the difference between two decades was not significant (p=0.187).

Comparing to the 1stD, significant variations in the composition of different pT-stages were seen in our study (p<0.001). To better understand the specific trends, we separately analyzed the incidence of MIUCs which has significantly decreased in the last 10 years (p<0.001). However, both the incidence of various histopathological grading of the renal pelvic tumors (p=0.385) and ureteral tumors (p=0.195) remained unchanged. Especially, the incidence of G3 tumors seemed not to change significantly between two decades (p=0.187). The evolutionary trend of pathological staging and grading depending on time were shown in **Figure 2**.

### Tumor Size

**Figure 3:**
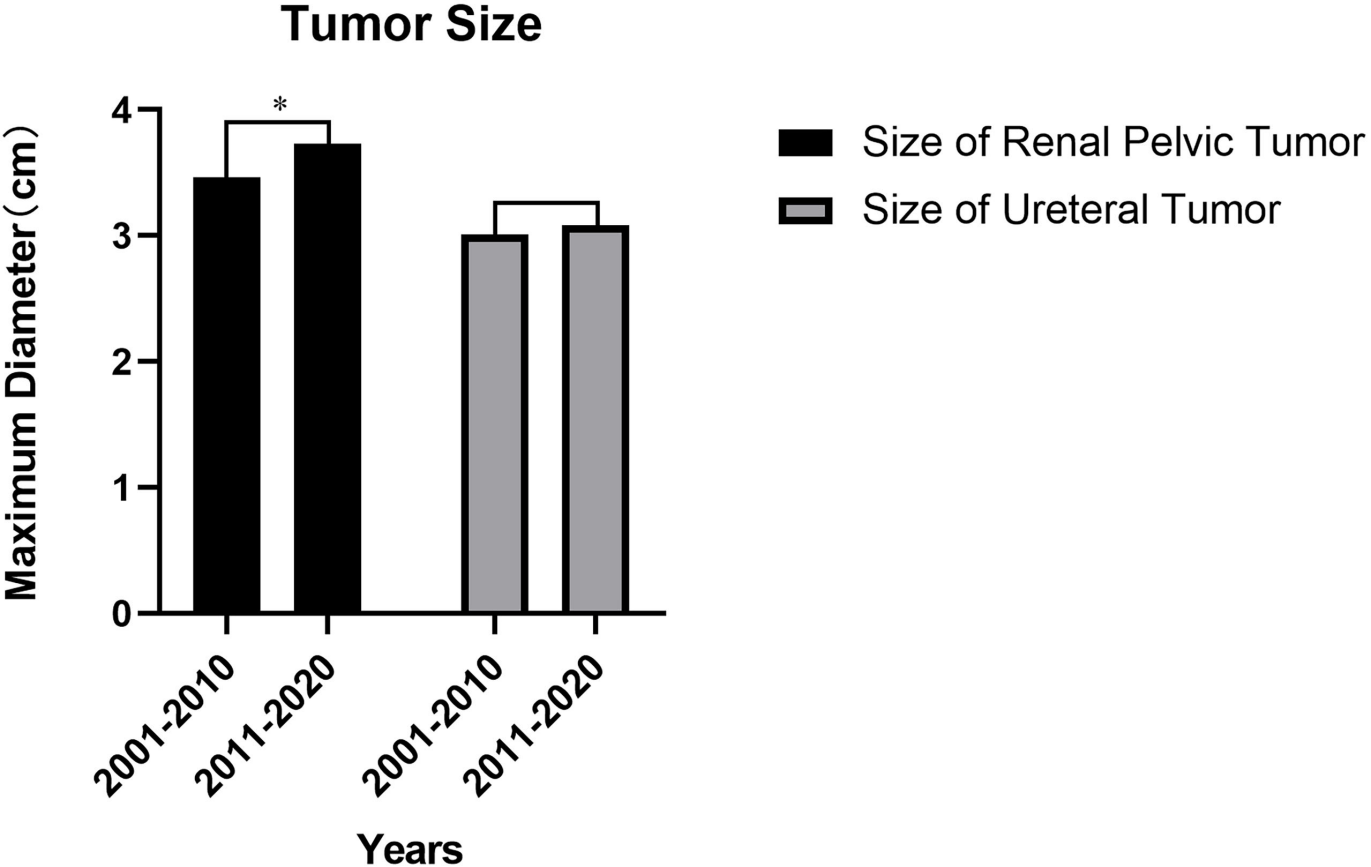
Tumor size of the UTUC in the 1st decade vs 2nd decade. *p=0.043.

Compared to 1stD, all UTUC patients in 2ndD had an increase in the mean maximum tumor diameter before the surgery. There was a significant difference in the increase in size of renal pelvic tumors (3.46 versus 3.73, cm, p=0.043, shown in **Figure 3**), however, ureteral tumors remained almost unchanged (p=0.609) (Detailed change about the tumor size was shown in **Tables 3-A** **,** **B**).

### Correlation Between Pathological Features and Prognosis

**Figure 4:**
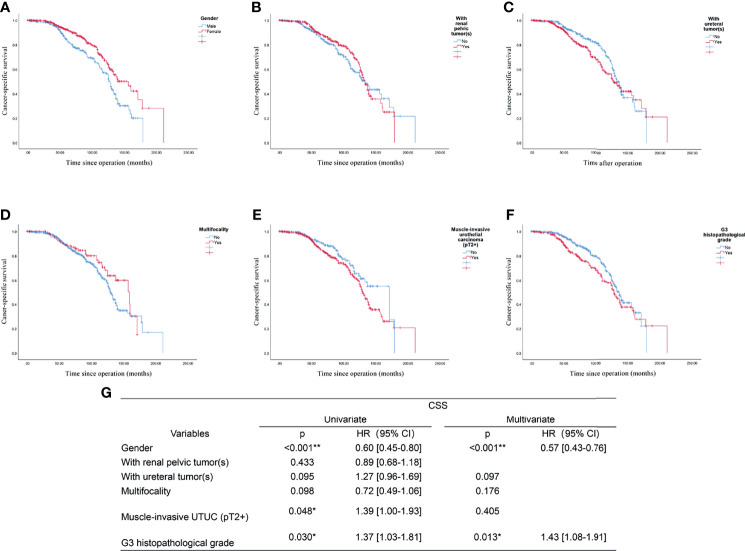
Prognostic analysis of the UTUC patients: the endpoint is cancer-specific survival, and the risk factor is: **(A)** Gender; **(B)** With renal pelvic tumor(s); **(C)** With ureteral tumor(s); **(D)** Multifocality; **(E)** Muscle-invasive urothelial carcinoma (pT2+); **(F)** G3 histopathological grade; **(G)** Univariate and multivariate analysis of all included factors. *p<0.05, **p<0.01.

The outcome of the univariate regression analysis suggested that male gender (p<0.001), muscle invasion (pT2+, p=0.048), and G3 histopathological grade (p=0.030) significantly predicted poorer CSS. In contrast, the tumor location and multifocality did not seem to affect the UTUC patients’ survival outcome significantly. After multifactorial Cox regression analysis, both male gender (p<0.001, HR: 0.57, 95%CI: 0.43-0.76), G3 histopathological grade (p=0.013, HR:1.43, 95%CI:1.08-1.91) might be two independent risk factors for predicting worse CSS in UTUC patients (shown in **Figure 4**) This finding indicated that though the histological grade of tumor did not changed significantly between past two decades, the increased incidence in male patients would suggest a relatively worse survival in UTUC than before.

## Discussion

Upper tract urothelial carcinoma (UTUC) is a sort of urological malignant tumor which performs relatively low incidence but poor prognosis. Because UTUC is often insidious, multicentric, and aggressive in origin, there may be a high risk of recurrence even after timely radical surgical treatment (7). Among the many influencing factors associated with prognosis, high TNM stage of the tumor, high histological grade (G3), lymph node metastasis, multifocality, male patients, and renal pelvis tumor are notably associated with a high mortality or recurrence rate (4, 8). What is more, the pathological features of the tumors also make sense to the protocol of treatment of the patients. Although radical nephroureterectomy (RNU) remains the current gold standard procedure for localized UTUC, a proportion of patients will lose the opportunity to receive adjuvant chemotherapy due to postoperative renal insufficiency (9). Patients with low-risk UTUC (unifocal disease, non-muscle invasion, pathologic biopsy, and urine cytology suggesting low histologic grading, maximum diameter <2 cm, and with no evidence of metastasis) have the opportunity to undergo the kidney-sparing surgery, which means they could have a comparable prognosis and better quality of life (1, 10, 11). Therefore, understanding the characteristics of the natural history and pathology plays a crucial role in predicting the prognosis of patients with UTUC. Unlike the Western population, the Chinese patients of UTUC have distinctive characteristics (12). Thus, clinical treatment guidelines and assessment of prognosis for Chinese UTUC patients are always difficultly obtained by totally copy the experience of European and American countries, which deserves exploration in the future. After analysis, our study suggested that the morbidity characteristics of Chinese UTUC patients were predominantly in the middle to high age group (55-70 years old), female, with ureteral tumors, muscle invasion (pT2+), and high-grade pathology (G2, G3), which was consistent with the outcome of Singla’s (13). With the advancement of science and technology, the treatment paradigm of UTUC has been updated (14), but as far as we know, there were no studies have been conducted on the evolution of the natural history and pathological features of this disease over the past 20 years. Munoz (15) once analyzed the evolution of the incidence and survival of UTUC in the United States from 1973 to 1996, according to the National Cancer Institute Surveillance, Epidemiology and End Results (SEER) database, and concluded that the incidence of UTUC has shown a mild increase and a gradual increase in 5-disease specific survival, and hypothesized that this trend might be related to the widespread use of ureteroscopy. Raman et al. (16) similarly found a slow increase in the overall incidence of UTUC. Unlike the above two researchers, Wu (3) suggested a decreasing trend in the incidence of UTUC in the United States and a predominance of male patients, renal pelvic tumors, and patients older than 70 years. And this conclusion revealed a gradual change in the trend of UTUC pathogenesis.

In the present study, we found a significant decrease in the incidence of renal pelvic tumors, muscle-invasive and multifocal UTUC in the last 10 years, but the histological grading of the tumors remained unchanged, however, the incidence of the ureter and the size of the renal pelvic tumors showed an increasing trend. It was reported that patients with UTUC who were aware of active self-monitoring and intervene in the disease before the onset of symptoms tended to have relatively less malignant pathological features and better prognosis (17). Thus, we speculated that the increase in the proportion of early-stage tumors may be related to the increased use of imaging examination technology such as CTU and the increased awareness of the population for self-monitoring. The evolution of tumor unifocal onset, histological grade, and tumor size may be related to the daily lifestyle habits of the Chinese population such as the application of aristolochic acid drugs or the changing disease spectrum of some chronic diseases such as lithanguria and chronic kidney disease (18–20). Further trials are needed to validate the conjecture.

To evaluate whether there are any changes in the age and gender composition of current Chinese UTUC patients, we explored the trends in the proportion of different genders and the evolution of the patients into young patients group (<55 years old) and elderly patients (≥70 years old) group according to the previous studies (21). The result revealed that the proportion of male patients and the average age gradually increased over the past 20 years, but the proportion of young and elderly patients did not change significantly. Since previous studies have shown a female majority in the Chinese UTUC population, unlike in Western countries, this may be associated with the prevalence of aristolochic acid-based herbs. The current decrease in the proportion of female patients may be related to the reduction in the addition of aristolochic acid component drugs by Chinese pharmaceutical companies. At the same time, the risk factor of smoking makes the proportion of male patients appear relatively higher. Notably, we also found that male UTUC patients had significantly poorer cancer-specific survival females. This increasing-proportion trend of the male gender implies that the potential health and economic burden of UTUC on society are also increasing, which warrants early attention and preventive measures.

Based on the pathological features, the molecular characteristics of UTUC are a current research hotspot in the field of diagnosis and treatment. Despite sharing a similar histological type, it is still controversial whether the molecular features of UTUC are equivalent to bladder cancer due to the variability in clinical presentation and genomics (22). From an early understanding of the molecular mechanism of Lynch-related UTUC with mutations in MMR genes, the second-generation sequencing technology has immensely improved the efficiency of exploration now (22, 23). The discovery of molecules such as FGFR-3 has led to advances in molecular diagnosis and immunotherapy of UTUC (24). The maturation of urine-based methylation detection technology also marks the upcoming era of molecular non-invasive detection in UTUC (25). Recently, Fujii successfully classified UTUC into five subtypes based on mutation type (26). This subtype classification system may be combined with pathological diagnosis prospectively to better assist in foreseeing the prognosis of UTUC patients and selecting appropriate strategies of chemotherapy or immunotherapy. However, most of the conclusions of molecular studies still need to be validated by further clinical trials. We also expect a discovery of molecules which with higher selectivity for UTUC.

Our study still has some limits. It was a retrospective analysis, so the selective bias was relatively inevitable. And we could not reasonably infer the causes of this evolutionary trend based on the current conclusion. Secondly, the data analysis stemmed from a single-center database, one of the largest urologic oncology clinical centers in China, which meant the representativeness of the findings still needs to be validated by further refinement of the multicenter study. Finally, we selected all patients with well-established follow-up outcomes for prognostic analysis, which meant the representativeness would not be satisfied. However, the missed visits were because the patients treated were from various regions all over China, which makes it hard to return to our center and finish the follow-up. However, this situation also demonstrated that the selection bias of our study was relatively low.

## Conclusion

In conclusion, UTUC in the Chinese population changed significantly in the last 20 years in patients’ age, gender composition, primary site, and multifocality. The proportion of high-T-stage UTUC was gradually decreasing and might imply an improved prognosis in general, but the size of the renal pelvic tumors seemed bigger, while there was no significant change in pathological grading. This trend may be related to the update of diagnostic techniques and self-monitoring awareness, which still needs multi-center trials to verify in the future.

## Data Availability Statement

The raw data supporting the conclusions of this article will be made available by the authors, without undue reservation.

## Ethics Statement

The studies involving human participants were reviewed and approved by Ethics Committee of Peking University First Hospital. Written informed consent for participation was not required for this study in accordance with the national legislation and the institutional requirements.

## Author Contributions

CX, CY, and CZ contributed equally to this article. CX was the first author and CY and CZ were the co-first author. JL, LQZ, and XL were the equally correspondent author of this article. All authors are accountable for all aspects of the work. Conceptualization: CX, CY, and DF. Formal analysis: CX, CY, and CZ. Investigation: CY, CX, ZL, and YW. Methodology: CX, CY, DF, XW, and XL. Project administration: LQZ, XL, CZ, and ZH. Resources: CY, YY, QT, GX, and LZ. Supervision: JL, XL, LQZ, and ZH. Visualization: CX, CY, and CZ. Writing – original draft: CX, CY, and CZ. Writing – review & editing: CX, CY, CZ, JL, LQZ, and XL. All authors approved the final article.

## Funding

This study was funded by Wuxi “Taihu Talents Program” Medical and Health High-level Talents Project and Beijing Municipal Science and Technology Commission Projects (Nos. Z171100001017093).

## Conflict of Interest

The authors declare that the research was conducted in the absence of any commercial or financial relationships that could be construed as a potential conflict of interest.

## Publisher’s Note

All claims expressed in this article are solely those of the authors and do not necessarily represent those of their affiliated organizations, or those of the publisher, the editors and the reviewers. Any product that may be evaluated in this article, or claim that may be made by its manufacturer, is not guaranteed or endorsed by the publisher.
